# Author Correction: Relatively warm deep-water formation persisted in the Last Glacial Maximum

**DOI:** 10.1038/s41586-026-10186-3

**Published:** 2026-01-27

**Authors:** Jack H. Wharton, Emilia Kozikowska, Lloyd D. Keigwin, Thomas M. Marchitto, Mark A. Maslin, Martin Ziegler, David J. R. Thornalley

**Affiliations:** 1 Department of Geography, University College London, London, UK; 2 Department of Earth Sciences, Utrecht University, Utrecht, Netherlands; 3 Woods Hole Oceanographic Institution, Woods Hole, MA USA; 4 Department of Geological Sciences and INSTAAR, University of Colorado, Boulder, CO USA

**Keywords:** Palaeoceanography, Physical oceanography, Palaeoclimate

Correction to: *Nature* 10.1038/s41586-025-10012-2 Published online 21 January 2026

In the version of the article initially published, the label “35.50” on the Fig. 1b colour scale bar should have read “35.00” and has now been corrected in the HTML and PDF versions of the article.

