# Supplementary material for: Relatively warm deep-water formation persisted in the Last Glacial Maximum
Source: Nature. 2026 Jan 21;650(8100):116–22. doi: 10.1038/s41586-025-10012-2 (PMC12872452; doi:10.1038/s41586-025-10012-2)
Supplement: Supplementary file 1 — Supplementary Information file containing scanning electron microscopy images of representative benthic foraminifera used for clumped isotope analysis; and an introductory section describing the relevant methods and main findings is followed by nine figures, each comprising four scanning electron microscopy images of monospecific specimens from a single core, accompanied by a caption describing the images. [file 41586_2025_10012_MOESM1_ESM.pdf]

nature portfolio

https://doi.org/10.1038/s41586-025-10012-2

Supplementary information

Relatively warm deep-water formation persisted in the Last Glacial Maximum

In the format provided by the authors and unedited

Supplementary Information: Relatively warm deep water formation persisted in the Last Glacial Maximum

To assess potential post-depositional alteration from authigenic carbonate precipitation and/or diagenetic overgrowths, representative glacial benthic foraminifera used for clumped-isotope (Δ_{47}) analysis were imaged using a scanning electron microscope (SEM). The resulting images are shown in Figures 1-9.

Samples were obtained following the same procedures as described in Methods, i.e., disaggregated mud was washed through a 63 µm sieve using deionized water, the coarse fraction was dried, and then the foraminifera picked from dry sieved size fraction, under a binocular light microscope using a wet (deionized water) fine brush. During picking, any visible adhering particles were gently removed with the wet fine brush. The foraminifera were not chemically cleaned or ultrasonicated, in order to keep their condition consistent with the specimens analysed for Δ_{47}, which also did not undergo such cleaning. Foraminifera were mounted on an aluminium stub using carbon tape and sputter-coated with ~10 nm of gold to improve conductivity. Imaging was carried out on a Jeol JSM-6480LV SEM at the Department of Earth Sciences, University College London, using an accelerating voltage of 7 kV and a working distance of 10 mm.

Overall, the SEM images show well-preserved foraminifera, with clearly defined ornamentation, whorls, sutures, open pores, and other surface features, and lack evidence of post-depositional alteration such as authigenic carbonate precipitation and/or diagenetic overgrowths, which is consistent with the independent geochemical evidence described in the main text.

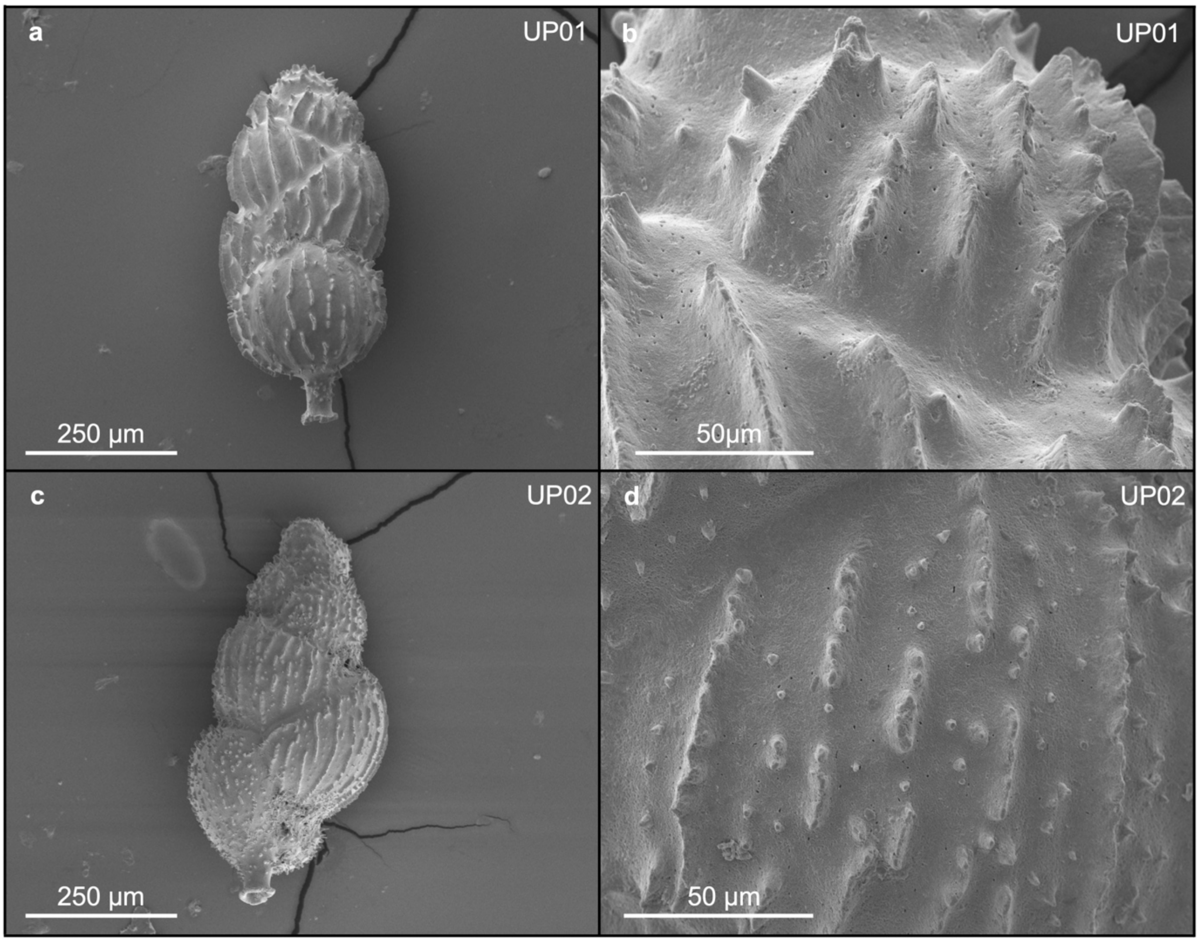

Figure 1. SEM images of individual *U. peregrina* from the glacial section of core ODP-172-1057 (1057-B-1-H-2, 58-88 cm core depth; 2.58 km water depth). Well-preserved whole specimens showing minimal signs of alteration, with clearly defined costae and sutures (**a**, **b**). Higher-magnification views of each specimens' costae reveal smooth intercostal surfaces and open pores between these features (**c**, **d**).

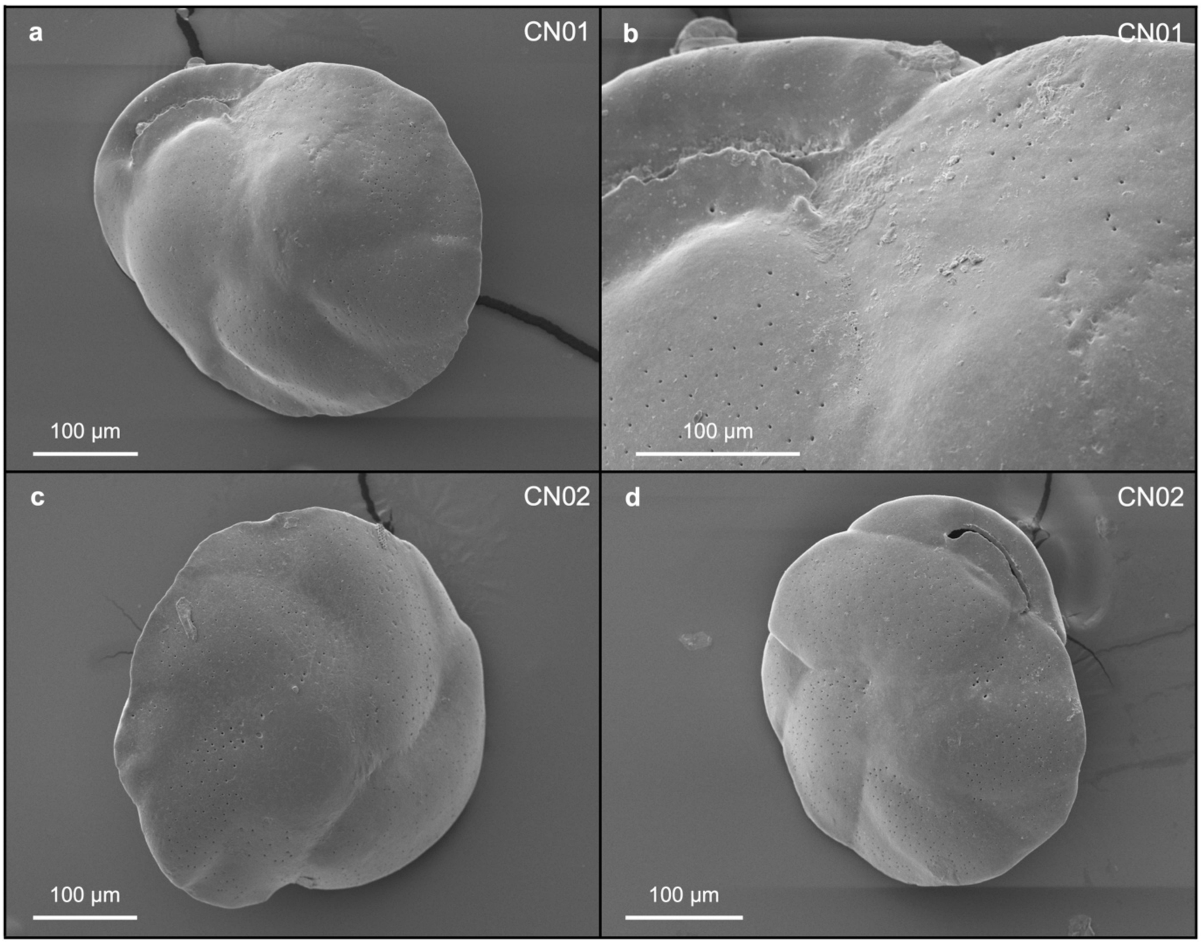

Figure 2. SEM images of individual *C. neoteretis* from the glacial section of core KNR-178-1-15JPC (635-690 cm core depth; 2.63 km water depth). Whole specimens are well-preserved and show negligible signs of alteration (**a**, **c** and **d**), with clearly defined alternating chambers and sutures, consistent with good preservation. Despite the occasional adhering clay particle, the test surfaces of each specimen are smooth, with open pores on the chamber walls and no pores on the umbilical boss. In panels **a** and **d**, the aperture of each specimen is visible, and panel **c** provides a higher-magnification view of the specimen in panel **a**, showing the aperture and surrounding pores in greater detail.

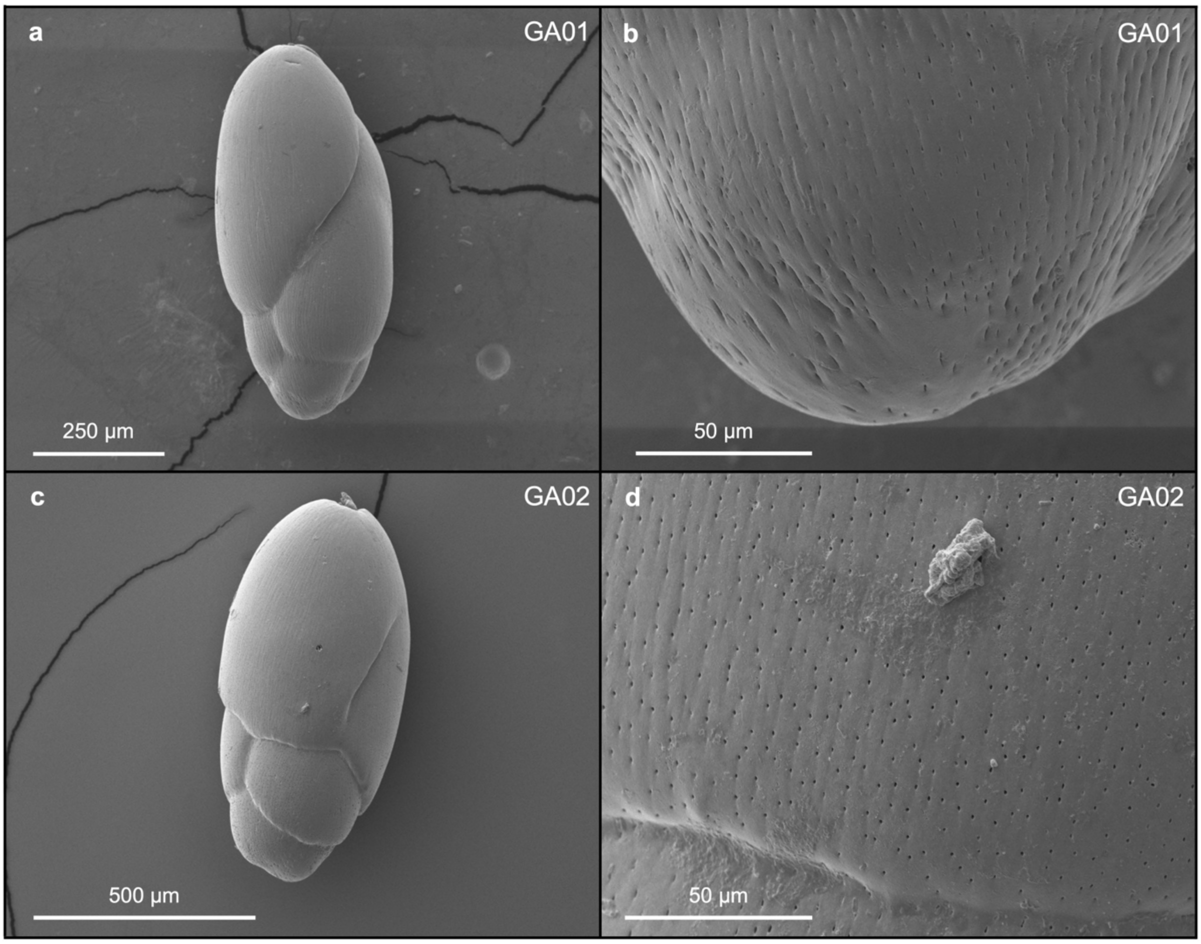

Figure 3. SEM images of individual *G. affinis* from the glacial section of core KNR-178-1-15JPC (620-735 cm core depth; 2.63 km water depth). Whole specimens (a, c) and higher-magnification views (b, d) reveal clearly visible sutures, striations, and open pores, which is consistent with good preservation.

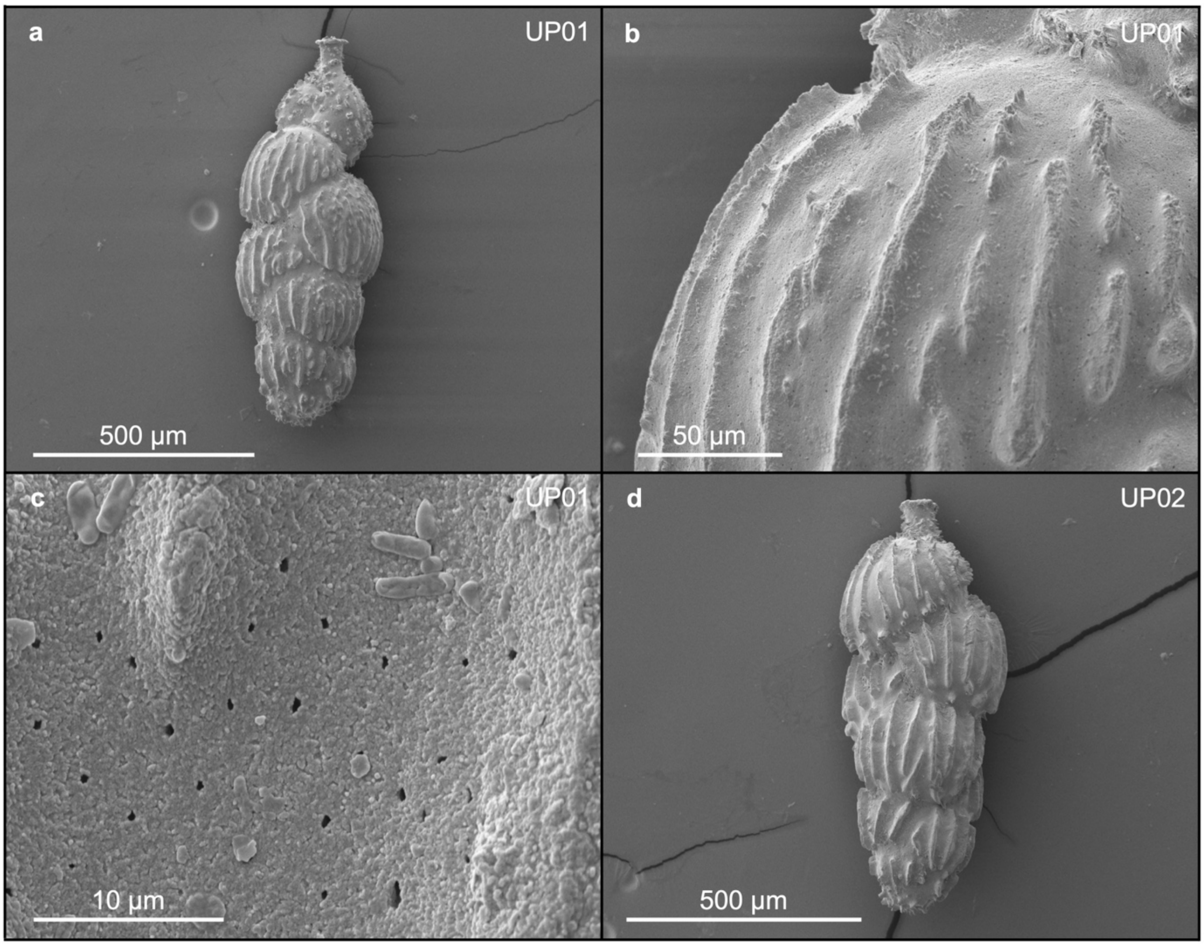

Figure 4. SEM images of individual *U. peregrina* from the glacial section of core KNR-178-1-15JPC (620-735 cm core depth; 2.63 km water depth). Whole specimens (**a**, **d**) are well preserved, showing clearly defined costae. Panels **b** and **c** present progressively higher-magnification views of the specimen in panel **a**, with panel **b** showing the costae in greater detail and panel **c** revealing evenly distributed open pores between each costa. At the highest magnification, occasional adhering clay particles are visible between the costae.

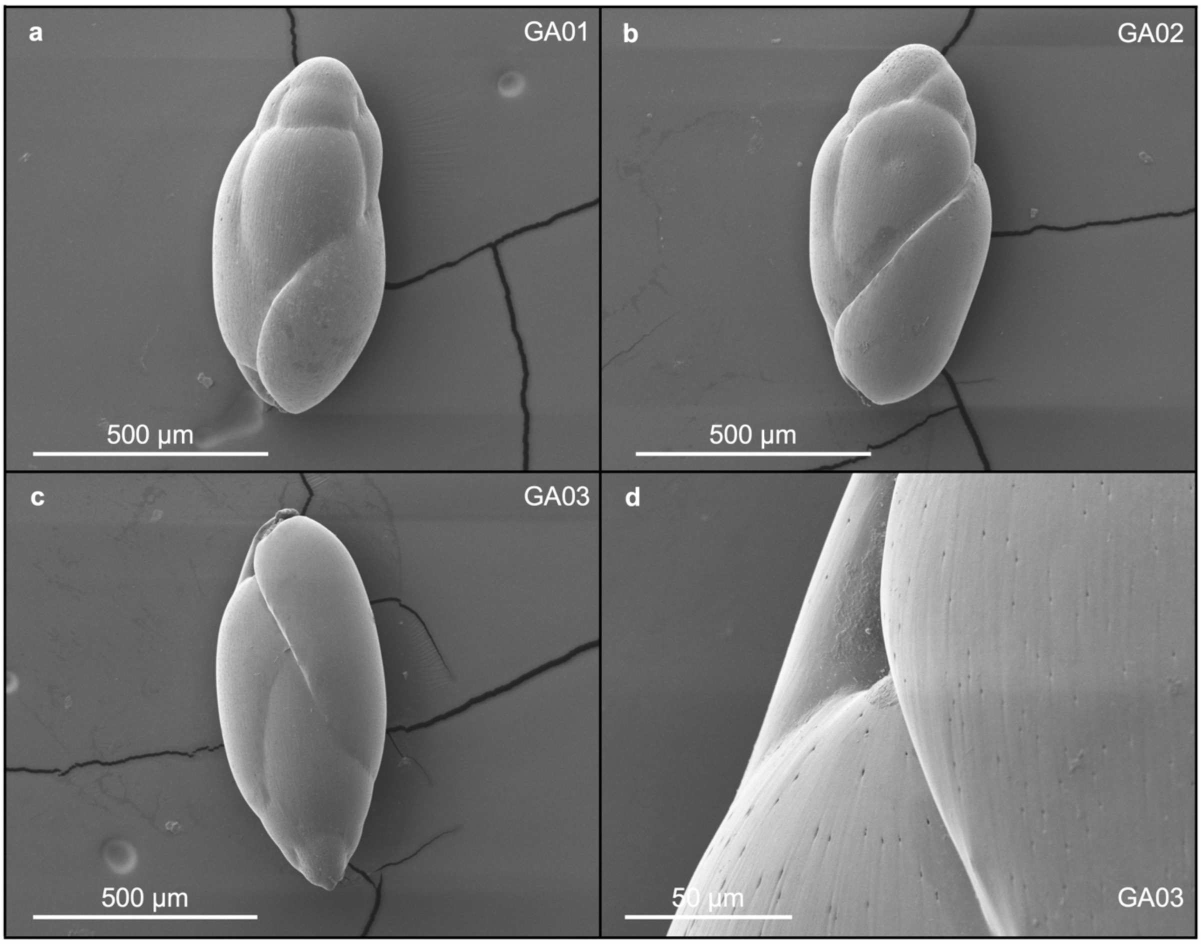

Figure 5. SEM images of individual *G. affinis* from the glacial section of core ODP-172-1059 (1059-B-1-H-4, 58-150 cm core depth; 3.00 km water depth). Whole specimens (**a**, **b** and **c**) show clearly defined chambers, sutures, striations, and apertures, indicating good preservation. Panel **d**, a higher-magnification image of the specimen shown in panel **c**, also suggests negligible contamination, displaying a smooth surface and revealing open pores situated along the striations.

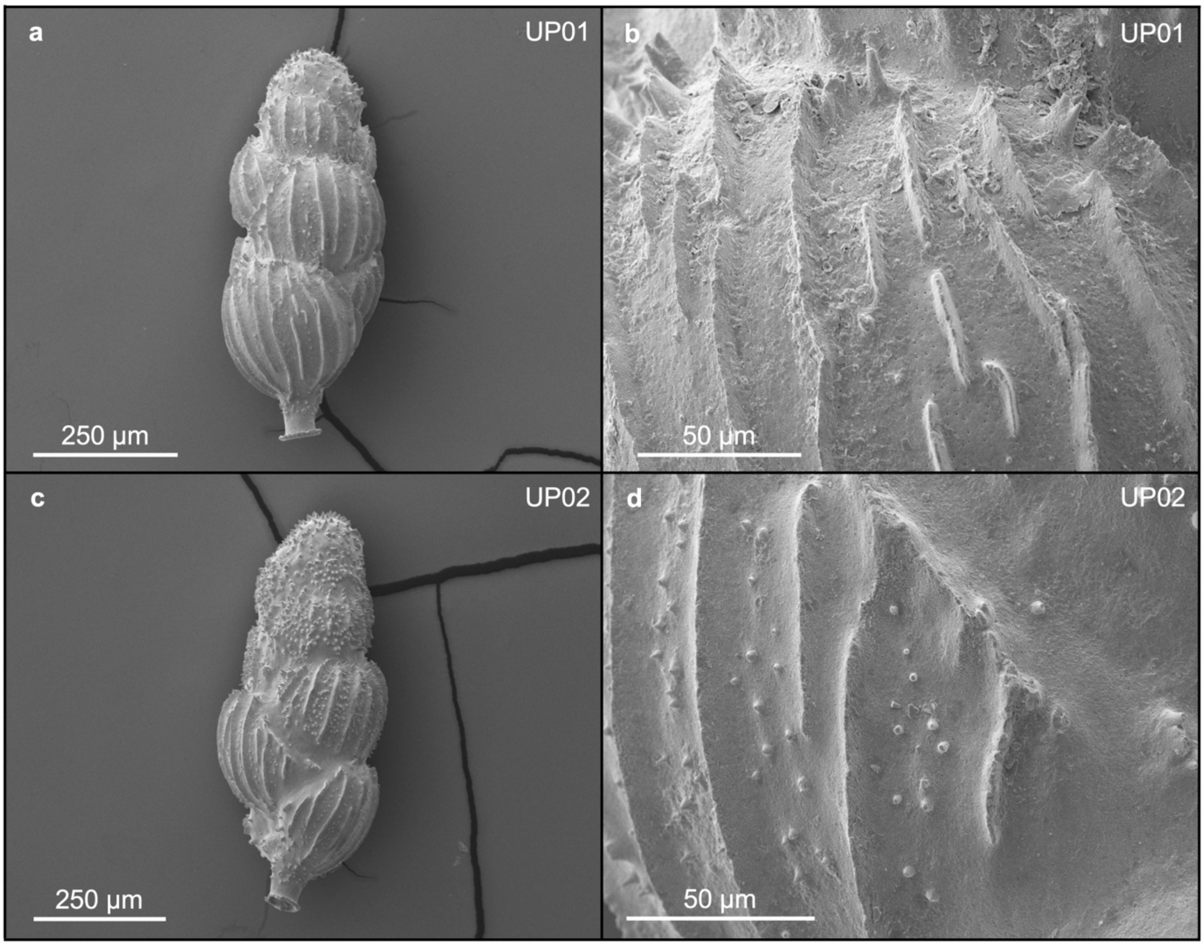

Figure 6. SEM images of individual *U. peregrina* from the glacial section of core ODP-172-1059 (1059-B-1-H-4, 58-150 cm core depth; 3.00 km water depth). Whole specimens with clearly defined chambers and costae are shown in panels **a** and **c**. Panels **b** and **d** present higher-magnification views of the specimens in panels **a** and **c**, respectively. Both higher-magnification images show well-preserved costae with no signs of alteration, although isolated adhering clay particles are visible on the specimen in panel **b**.

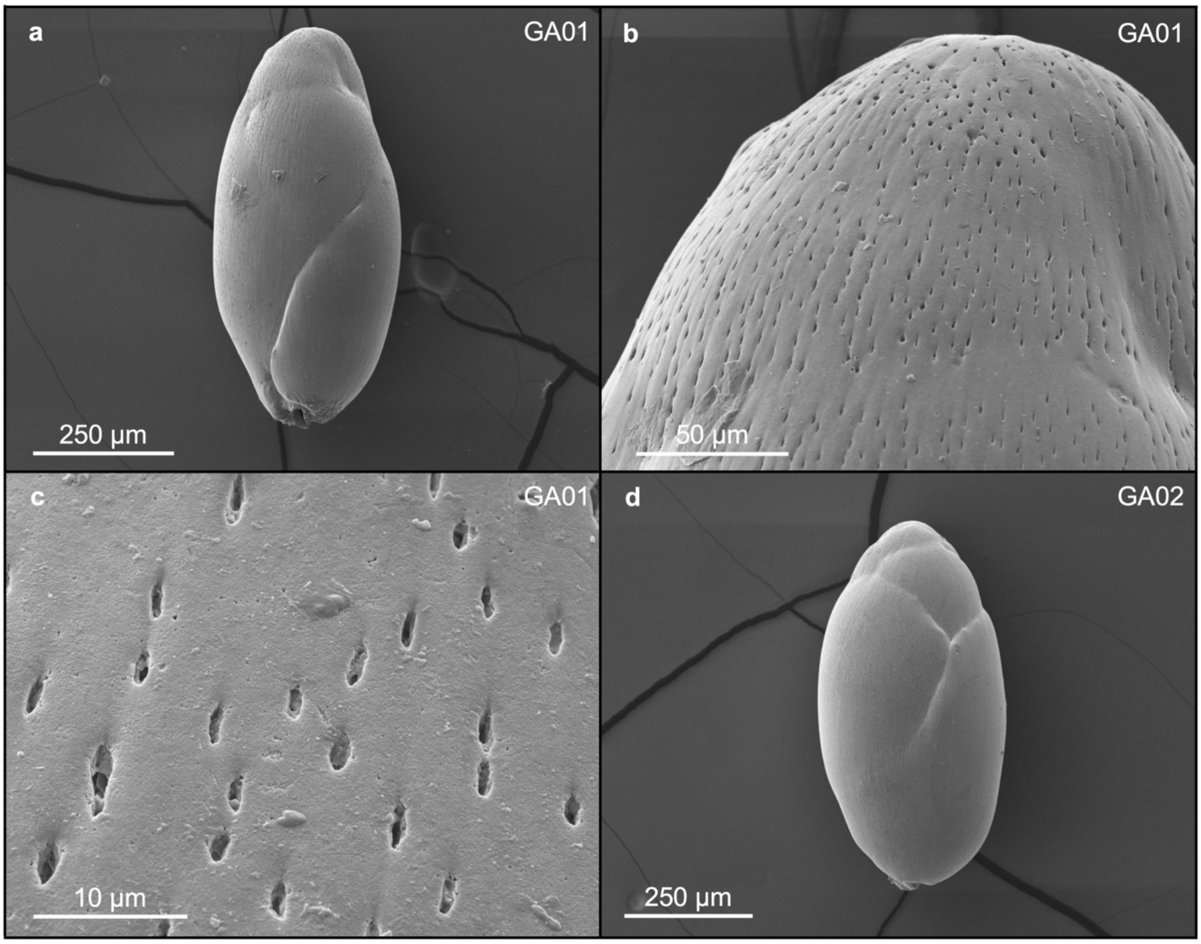

Figure 7. SEM images of individual *G. affinis* from the glacial section of core KNR-178-1-10JPC (560-700 core depth; 3.02 km water depth). Whole specimens with clearly defined sutures and striations are shown in panels **a** and **d**. Panel **b** is a higher-magnification view of the specimen in panel **a**, revealing a smooth test surface and open pores punctuating the striations, consistent with good preservation. Panel **c** presents an even higher-magnification image of the same specimen, highlighting the well-preserved open pores.

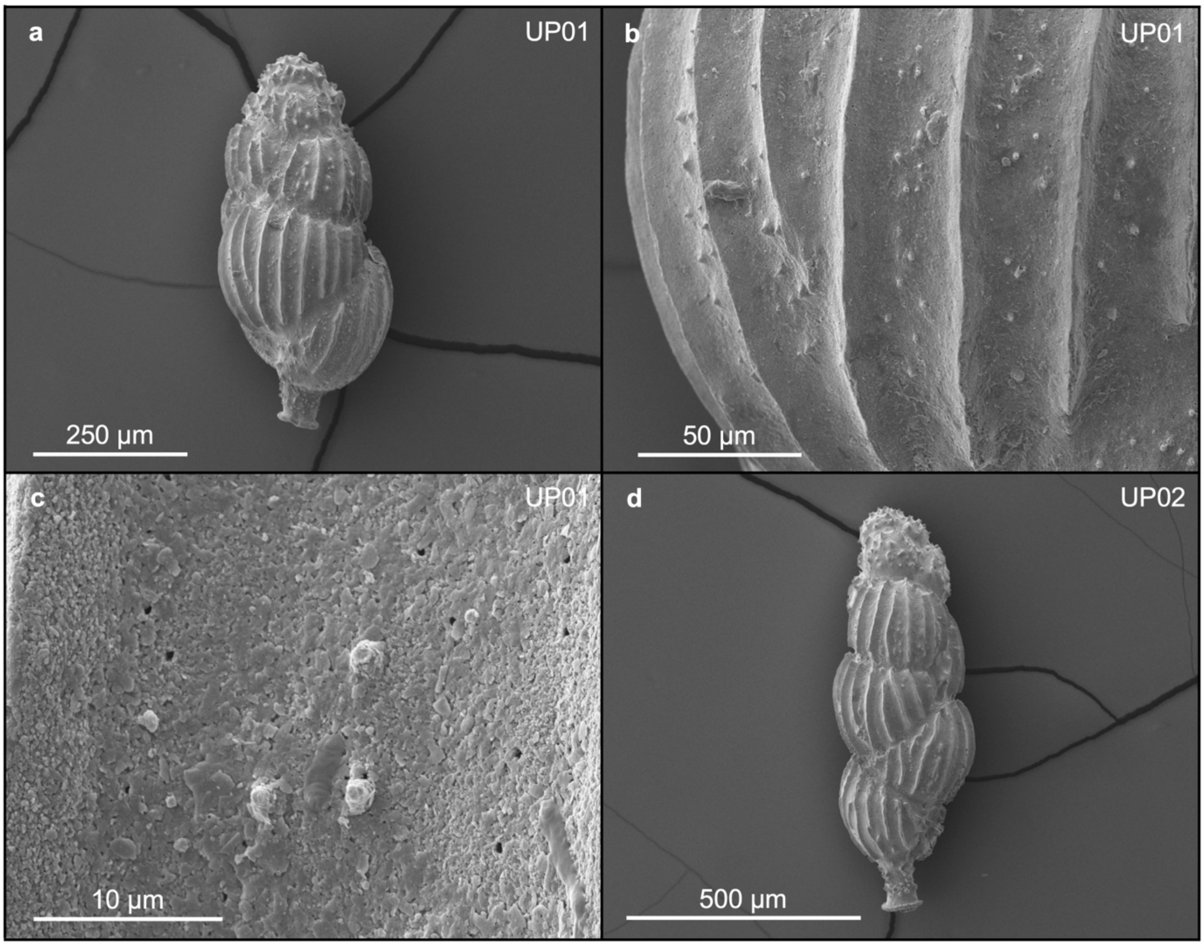

Figure 8. SEM images of individual *U. peregrina* from the glacial section of core KNR-178-1-10JPC (560-700 core depth; 3.02 km water depth). Well-preserved whole specimens with clearly defined chambers and adorning costae are shown in panels **a** and **d**. Panel **b** is a higher-magnification image of the costae of the specimen in panel **a**, and panel **c** presents an even higher-magnification view of the same specimen, revealing open pores, with occasional adhering clay particles also visible.

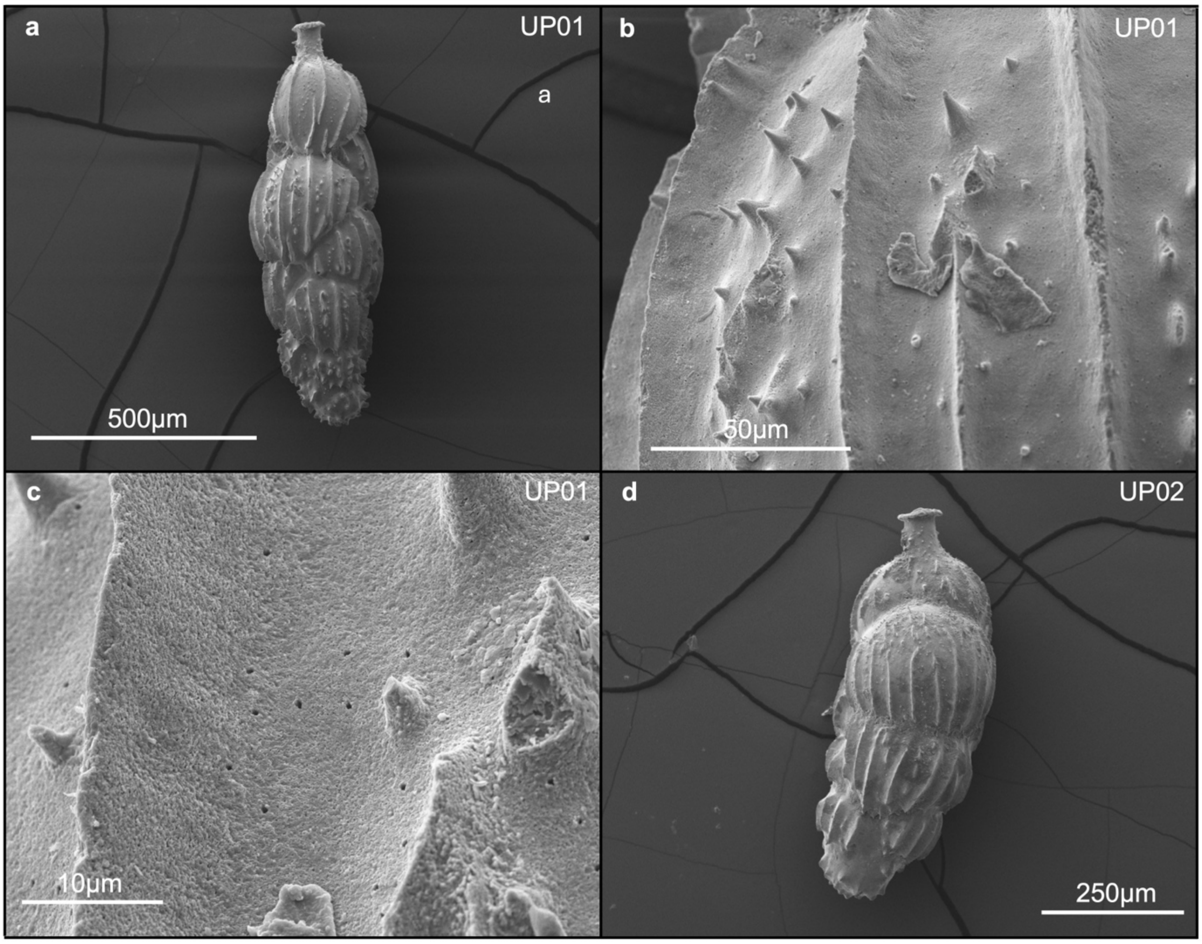

Figure 9. SEM images of individual *U. peregrina* from the glacial section of core KNR-178-1-2GGC (210-235 core depth 3.93 km water depth). Whole specimens show negligible signs of alteration, with very well-defined costae (**a**, **d**). Panel **b** is a higher-magnification image of the specimen shown in panel **a**, illustrating the well-preserved costae, as well as isolated adhering clay particles. Panel **c**, an even higher-magnification view of the same specimen, reveals clearly defined open pores indicative of good preservation.
